# Signal weighting as an organizing principle of adaptive systems

**DOI:** 10.1093/pnasnexus/pgag333

**Published:** 2026-10-01

**Authors:** Duncan Bruce Sutherland, Bruno Lemaitre

**Affiliations:** School of Architecture, Civil and Environmental Engineering (ENAC), École Polytechnique Fédérale de Lausanne (EPFL), 1015 Lausanne, Switzerland; Global Health Institute, School of Life Sciences, École Polytechnique Fédérale de Lausanne (EPFL), 1015 Lausanne, Switzerland

**Keywords:** adaptive systems, signal weighting, host-microbiome interactions

## Abstract

Current biological frameworks centered on replication, variation, and selection provide powerful explanations for evolutionary change but do not fully explain how adaptive systems determine relevance in context or maintain coordinated behavior across interacting components. Across diverse domains—including immune recognition, host–microbiome interactions, neural systems, and ecological regulation—adaptive behavior depends on the integration, prioritization, and modulation of signals through feedback. Here, we propose signal weighting as a conceptual framework for understanding how adaptive systems assign, update, and stabilize relevance over time. Signal weighting describes the context-dependent modulation of signal influence through processes that filter, amplify, attenuate, and recursively adjust responses based on prior interactions and system state. Importantly, adaptive systems rarely function in isolation. When signal-weighting processes become coupled across interfaces between interacting systems, their dynamics can stabilize coordinated behaviors that extend beyond individual components, contributing to the emergence of higher-order adaptive units. This logic is evident in immune pattern recognition, where signals are selectively reinforced or attenuated to maintain functional balance, and in host–microbiome systems, where immune and microbial processes reciprocally shape one another through continuous feedback across shared interfaces. In this view, adaptive organization emerges not only through selection acting on variation but also through the recursive stabilization of interactions that remain coherent under perturbation. By identifying shared weighting architectures across biological scales and substrates, signal weighting provides a conceptual bridge across fragmented adaptive literatures and offers a general organizational framework for understanding how coordinated complexity emerges, stabilizes, and persists in living systems.

## Adaptive systems and the problem of relevance

Adaptive systems operate in environments rich with signals. At every moment, biological systems are exposed to a multitude of inputs—molecular cues, environmental fluctuations, and interactions with other organisms—yet only a subset of these signals influences behavior. The ability to distinguish which signals matter and to respond appropriately is fundamental to survival across all forms of life. Despite its centrality, this problem of relevance—how systems determine which inputs are meaningful in a given context—remains only partially addressed by existing biological frameworks.

Classical evolutionary theory has provided a powerful account of how adaptive traits emerge over time through variation, selection, and inheritance (1, 2). These principles explain how populations change across generations and how advantageous features become established. However, these frameworks primarily address adaptation across generations and do not always directly address how adaptive responses are generated and regulated within organisms. As Waddington emphasized, understanding adaptation also requires mechanistic explanations linking developmental processes to interactions with the environment (3). Subsequent advances in systems biology, neuroscience, control theory, and related fields have expanded this perspective by describing adaptive behavior as a continuous process of context-dependent information integration (4, 5). In such settings, the challenge is not only to generate variation but also to interpret incoming signals, prioritize among competing inputs, and adjust behavior dynamically.

This challenge is particularly evident in systems where responses must be both flexible and constrained. Immune systems, for example, must detect and respond to potentially harmful signals while avoiding inappropriate activation (6). This requires not only the recognition of specific molecular patterns but also the integration of contextual cues that modulate the strength and nature of the response. Similarly, in host–microbiome interactions, organisms coexist with dense and dynamic microbial communities, maintaining functional stability despite constant perturbation (7). In these systems, adaptive behavior depends on ongoing evaluation of signals and the maintenance of balanced interactions rather than on discrete selection events alone.

Across such examples, a common feature emerges: adaptive systems do not respond to signals in a binary or fixed manner but continuously adjust the relative importance of different inputs. Signals are filtered, amplified, or suppressed depending on context, history, and system state. This dynamic process enables organisms to maintain stability while remaining responsive to change, supporting both robustness and adaptability.

Current frameworks in biology capture many aspects of these processes, including molecular recognition, regulatory networks, and feedback control (4). However, these descriptions are often domain-specific and do not provide a general account of how relevance is assigned across different types of adaptive systems. As a result, related processes of contextual signal prioritization have been described independently across diverse biological domains—including immune recognition, neural integration, developmental canalization, ecological regulation, and receiver bias in sexual selection—without being treated as manifestations of a shared organizational logic (8, 9).

Existing theoretical frameworks—including predictive processing, control theory, reinforcement learning, and the free energy principle—have generated important mechanistic insights into adaptive behavior (5, 10–12). Here, we ask a complementary question: whether these diverse mechanisms instantiate a common minimal causal architecture through which adaptive systems determine relevance, integrate signals, stabilize interactions, and generate higher-order organization across biological domains.

Importantly, adaptive systems rarely function in isolation. Across biological contexts, signals are exchanged and interpreted at interfaces between interacting systems—including host and microbiome, immune and metabolic networks, cells within tissues, and organisms within ecological communities. The stabilization of interactions across such interfaces may represent a recurrent organizational feature underlying adaptive integration across scales.

In the following sections, we develop this perspective and propose signal weighting as a framework for understanding how adaptive behavior is structured, stabilized, and coordinated across biological systems.

## Adaptive processes beyond replication alone

The modern synthesis of evolutionary biology has placed replication, variation, and selection at the center of our understanding of life (1, 2, 13). This framework has been extraordinarily successful in explaining how adaptive traits arise and spread across populations over time. By linking heritable variation to differential reproductive success, it provides a coherent account of how complex biological features can emerge without the need for foresight or design. As such, replication-centric explanations remain foundational to biology (2).

However, many adaptive processes unfold on timescales and organizational levels that are not always directly addressed by replication-selection frameworks alone (4). Within organisms, responses to environmental and internal signals must be generated continuously, often in the absence of genetic change (3). These processes rely on mechanisms that interpret and regulate incoming information in real time (5), shaping behavior through dynamic adjustment rather than through selection across generations.

The immune system provides a clear example of this distinction. While its components are shaped by evolutionary processes, immune responses themselves depend on the context-dependent interpretation of signals (14). Pattern recognition receptors detect conserved molecular features, but the outcome of this detection is not fixed (15). Instead, it depends on a range of additional inputs, including signal strength, temporal dynamics, and the presence of co-stimulatory or inhibitory cues (16, 17). The system must discriminate between harmful and harmless signals, often under conditions of uncertainty, and calibrate its response accordingly (18). Although shaped by evolutionary processes, this adaptive behavior also depends on ongoing evaluation and modulation of relevance within the organism.

Similar dynamics are evident in host–microbiome systems. Hosts are continuously exposed to vast and diverse microbial communities, many of which contribute to physiological function (7, 19). The stability of these systems does not arise from the elimination of variation but from the continuous regulation and stabilization of interactions across host–microbiome interfaces (20). Immune and metabolic processes interact with microbial populations to maintain functional balance, allowing for both persistence and change (21). Here again, adaptation depends on the capacity to manage and stabilize relationships rather than simply to select among discrete variants (22).

Even at the level of cellular regulation, biological systems exhibit behavior that extends beyond replication-centric descriptions. Gene expression, signaling pathways, and metabolic networks respond to fluctuating conditions through feedback mechanisms that adjust system activity in real time (5). These processes determine how information is processed and acted upon within cells, influencing outcomes independently of changes in genetic sequence (23).

Taken together, these examples suggest that while replication and selection explain how adaptive features are established and propagated, they do not fully describe how adaptive behavior is generated and maintained within systems. In particular, they offer limited insight into how systems determine which signals to act upon, how competing inputs are prioritized, and how stable patterns of interaction emerge from continuous feedback.

Recognizing these limitations does not diminish the importance of evolutionary theory but highlights the need for complementary perspectives that address the organization of adaptive processes at different scales. By focusing on how systems interpret and regulate signals, it becomes possible to identify organizational principles that operate alongside replication and selection to shape adaptive behavior across biological scales.

In the next section, we examine these processes more directly and propose signal weighting as a framework for understanding how adaptive systems assign relevance, integrate information, and stabilize adaptive interactions across biological contexts.

## Signal weighting in biological systems

Adaptive behavior requires more than the detection of signals; it depends on how signals are interpreted and acted upon in context. Across biological systems, responses are rarely determined by single inputs in isolation. Instead, multiple signals are integrated, compared, and modulated to produce outcomes that are appropriate to the current state of the system. This process involves not only recognizing signals but also assigning them relative importance.

We use the term “signal weighting” to denote this abstract adaptive operation—the context-dependent assignment of differential causal influence to relevant signals. Weighting is not itself a specific biochemical mechanism but an abstract operation implemented through diverse processes—including receptor affinities, signaling thresholds, synaptic plasticity, regulatory interactions, ecological feedbacks, or evolutionary selection. In each case, weighting determines the relative contribution of competing signals to subsequent system behavior. Signal weighting refers to the dynamic assignment of context-dependent causal influence to relevant signals through mechanisms that filter, amplify, or suppress their influence on system behavior. Rather than responding in a binary manner, adaptive systems continuously adjust the contribution of different inputs, allowing responses to be shaped by context, history, and feedback. Unlike rigid feedback mechanisms in which responses are largely predefined through fixed regulatory loops that maintain set points, as in classical control systems, signal weighting enables systems to continuously re-adjust the relative influence of signals based on context and prior states.

A more detailed operational description of this architecture is provided in Supplementary Note S1. This process can be represented as a minimal recursive architecture in which signals are evaluated, acted upon, and updated through feedback (Fig. 1).

**Figure 1:**
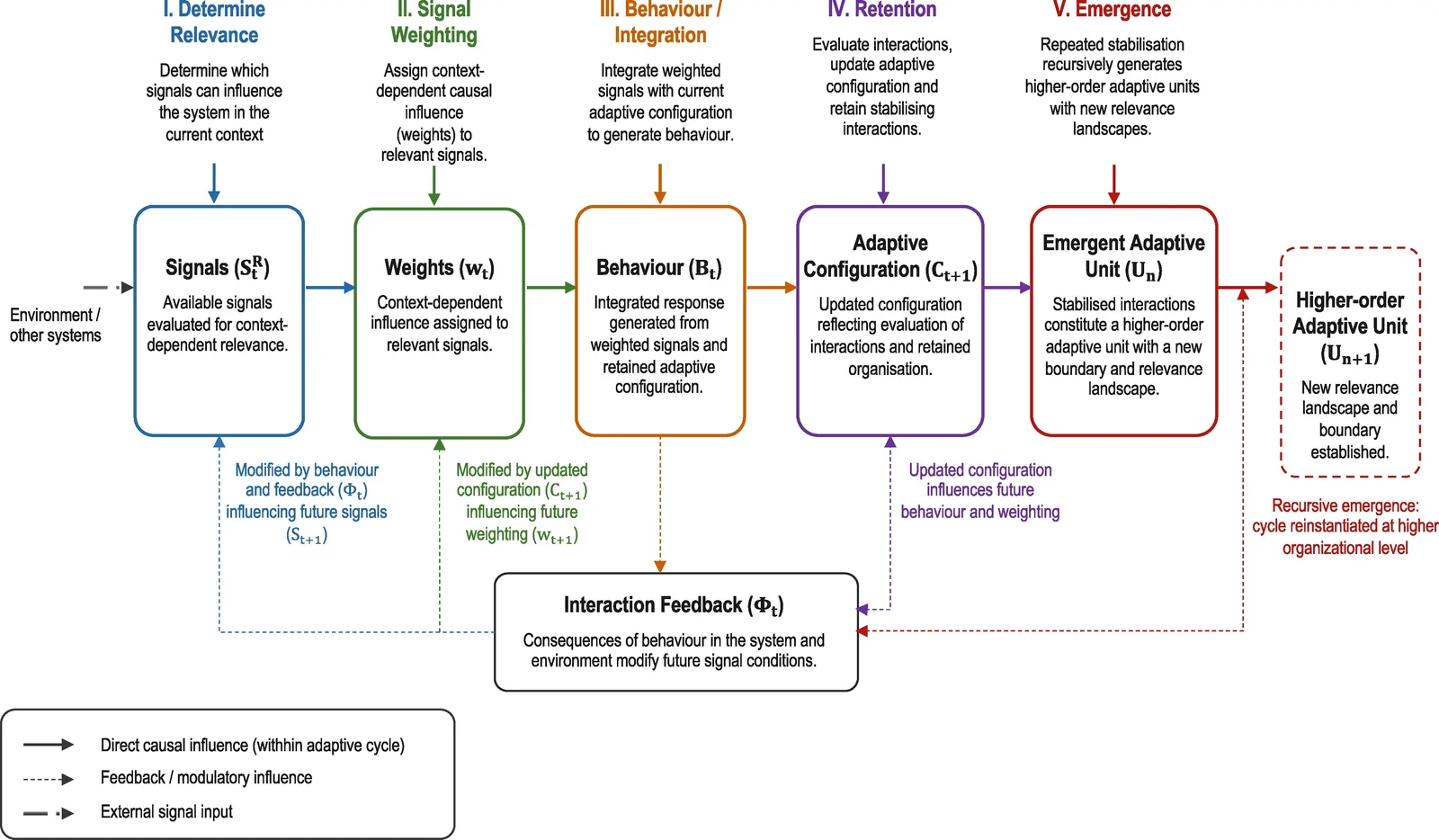
Recursive causal architecture of SWT. SWT describes adaptation as a recursive causal architecture rather than a linear sequence of independent operations. Available signals (S) are evaluated in relation to system state (x) and interaction history (h), determining their context-dependent weights (w). Weighted signals are integrated with the current adaptive configuration (C) to generate behavior (B). Behavior alters interactions with the system and its environment, generating feedback (Φ) that updates adaptive configuration and influences subsequent signals, thereby closing the adaptive loop. Configurations associated with continued functional coherence are preferentially retained (U), allowing prior interactions to modify future signal weighting and behavior. Repeated stabilization of coupled interactions can generate higher-order adaptive units, establishing new system boundaries and relevance landscapes in which the same causal architecture may recur. The five SWT principles therefore define an ordered set of adaptive operations whose causal dependencies form a recurrent rather than strictly linear architecture.

This logic is particularly evident in the immune system. Pattern recognition receptors detect conserved molecular motifs associated with pathogens or tissue damage, but the downstream response depends on more than detection alone (14). The same molecular signal can lead to activation, tolerance, or suppression depending on its context—for example, its concentration, persistence, or co-occurrence with other signals. Cytokine environments, cellular states, and prior exposures all influence how signals are interpreted, effectively weighting their contribution to the overall response (17). Through these mechanisms, the immune system maintains a balance between responsiveness and restraint, adapting its behavior to complex and changing conditions (see Supplementary Note S2 for a detailed mechanistic treatment). This adaptive capacity is further illustrated by innate immune priming, in which prior exposure to a stimulus enhances the speed or magnitude of subsequent responses (24). Such priming reflects a retained bias in signal interpretation, consistent with signal weighting processes that update and stabilize relevance based on prior interactions. Similar principles are evident in the nervous system, where neurons integrate multiple inputs through weighted synaptic connections (Supplementary Note S4). The relative influence of these inputs is not fixed but dynamically adjusted through activity-dependent plasticity, effectively reweighting inputs based on experience (25).

Signal weighting also operates at the level of host–microbiome interactions. Hosts are exposed to continuous streams of microbial signals derived from commensal and transient organisms (19). Rather than mounting uniform responses, host systems selectively modulate their interactions with these signals, promoting tolerance in some contexts and defense in others (21). Microbial communities, in turn, respond to host-derived signals, adjusting their composition and activity (7). The resulting system reflects ongoing feedback in which signals are not simply transmitted but are differentially reinforced or attenuated over time (Supplementary Note S3). Stability emerges not from the absence of variation but from the regulated weighting and stabilization of interactions across host–microbiome interfaces.

At the cellular level, similar principles can be observed in regulatory and signaling networks (26). Cells process inputs from multiple pathways, integrating signals related to nutrient availability, stress, and intercellular communication (23). The outcome of this integration depends on how these inputs are weighted within the network, influencing gene expression, metabolic activity, and cell fate decisions. Feedback loops play a central role in this process, allowing systems to adjust their sensitivity and recalibrate their responses based on prior states.

Across these examples, signal weighting provides a common description of how adaptive behavior is structured. It captures the idea that systems do not merely respond to signals but actively shape their responses by modulating the influence of different inputs. This process enables flexibility while maintaining coherence, allowing systems to adapt without losing functional stability. In this view, systems can be distinguished by their interpretive depth—the number and diversity of signals that can be meaningfully integrated and weighted—reflecting their capacity to generate context-sensitive responses across varying conditions.

We refer to this framework as signal weighting theory (SWT). Expanded illustrations across immunology, host–microbiome systems, neural systems, and Darwinian evolution are provided in Supplementary Notes S2–S5, with the core architecture described in Supplementary Note S1. In this view, adaptive systems can be understood in terms of how they assign, update, and stabilize the relevance of signals over time. While the specific mechanisms differ across biological contexts, the underlying logic—context-dependent weighting of inputs through feedback—is shared. By focusing on this process, it becomes possible to compare and relate diverse adaptive systems using a common conceptual language. Importantly, while the mechanisms described here are well established within their respective domains, their interpretation as instances of a shared signal-weighting process provides a unifying organizational framework that is not explicitly captured by existing domain-specific or system-level descriptions. From this perspective, Darwinian evolution may also be interpreted as a distinct implementation of the same organizational architecture, in which signal weighting is externalized through differential retention across generations rather than through real-time adaptive modulation within individual systems (Supplementary Note S5).

In the next section, we extend this perspective by considering how signal-weighting processes interact. When multiple adaptive systems are coupled, their respective weighting dynamics can influence one another, giving rise to stabilized patterns of behavior that extend beyond individual components.

## Coupling and stabilization of adaptive behavior

Adaptive systems rarely operate in isolation. Within biological contexts, multiple systems that assign and update signal relevance are continuously interacting, each influencing the behavior of the others (27, 28). When such signal-weighting processes are coupled, their dynamics can become interdependent, giving rise to coordinated and stabilized patterns of behavior that extend beyond individual components (Fig. 2).

**Figure 2:**
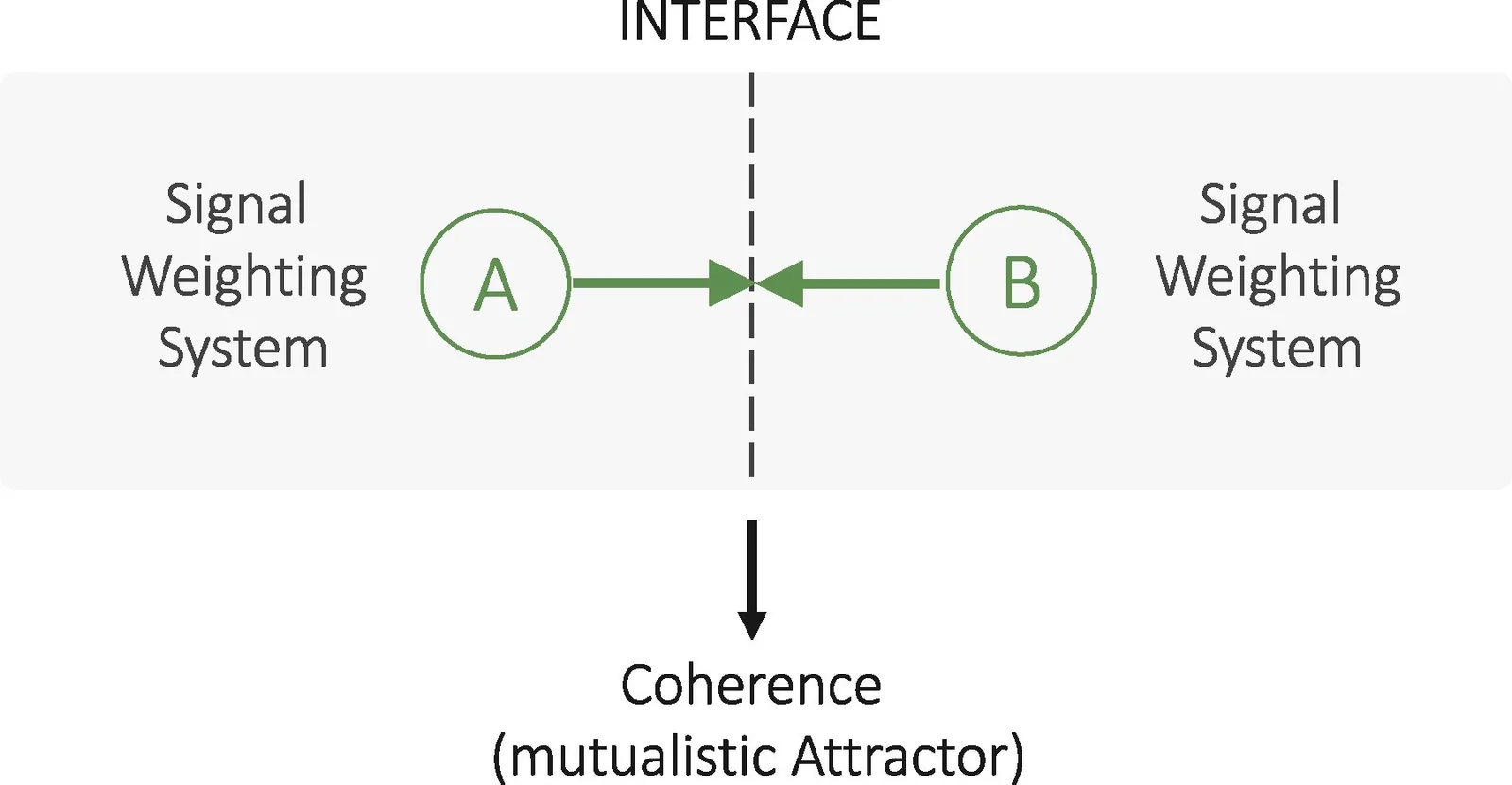
Signal-weighting interfaces drive integration and stabilized adaptive organization. Adaptive systems couple through interfaces that weight and stabilize signals between them. When such interfaces bias interactions toward mutual compatibility, coupled systems can converge on shared stable configurations (mutualistic attractors) that are selectively retained over time. In SWT, directionality in adaptive systems emerges from the stabilization of interactions at interfaces rather than from isolated system optimization.

Coupling occurs when the output of one system becomes an input for another, creating feedback loops that link their respective responses (28). In these settings, signal weighting is no longer confined to a single system but is distributed across interacting networks. The resulting behavior reflects not only the internal dynamics of each system but also the structure of their interactions. Over time, these coupled processes can reinforce particular patterns of activity, while dampening others, leading to the emergence of stable configurations. Moreover, this perspective emphasizes co-adaptive dynamics at shared interfaces between systems, rather than focusing solely on internal stabilization or the neutralization of external perturbations.

Host–microbiome systems provide a clear illustration of this principle, particularly through the role of immunoglobulin A (IgA) in shaping microbial communities (Supplementary Note S3). The host immune system continuously evaluates microbial signals, modulating responses that influence microbial composition and activity, with IgA acting as a key mediator of this interaction (29, 30). IgA does not exclude microbes uniformly but selectively binds subsets of the microbiota, promoting immune exclusion of some while supporting the retention and spatial organization of others, thereby influencing their persistence and metabolic activity. In turn, microbial communities produce metabolites and signaling molecules—such as short-chain fatty acids—that can regulate IgA responses and broader host physiology (28). This reciprocal exchange creates a tightly coupled system in which both host and microbial processes participate in shaping overall behavior, consistent with perspectives that emphasize the central role of symbiosis in animal biology (31). Stability is maintained not through static equilibrium, but through the ongoing adjustment of signal weighting across the interface. Perturbations—such as infection, dietary change, or antibiotic exposure—can shift this balance (32), but the system often returns to a functional state through the reestablishment of coordinated interactions.

Similar forms of coupling are observed within multicellular organisms. Related principles are discussed in neural systems (Supplementary Note S4). Cellular populations communicate through signaling molecules, forming networks in which the behavior of individual cells depends on the collective dynamics of the group (33). Feedback between cells can stabilize differentiation states, maintain tissue organization, and coordinate responses to external stimuli. In these systems, adaptive behavior emerges from the interaction of multiple signal-weighting processes operating at different levels of organization.

Coupling also allows for the integration of distinct functional domains. For example, metabolic and immune processes are increasingly recognized as interconnected, with metabolic states influencing immune responses and vice versa (28). Signals related to nutrient availability, metabolic state (e.g. vitamin-derived signals such as retinoic acid), and cellular stress are incorporated into immune decision-making, while immune activity can reshape metabolic pathways. These interactions further illustrate how adaptive behavior is shaped by the coordinated weighting of signals across system boundaries.

Across these examples, a common pattern emerges: when signal-weighting systems interact, configurations that support mutual stability tend to persist, while destabilizing interactions are attenuated or reorganized. This does not imply that systems move toward a fixed or optimal state, but rather that they favor arrangements in which coordinated behavior can be maintained despite ongoing perturbation. In this sense, stability is an emergent property of coupled signal-weighting processes, arising from the continuous adjustment of interactions rather than from static control mechanisms.

By focusing on coupling, signal weighting can be extended from a description of individual system behavior to a framework for understanding how higher-order organization emerges. Adaptive systems can be seen as networks of interacting processes that collectively assign and stabilize relevance across multiple scales. This perspective highlights the importance of interfaces—points of interaction where signals are exchanged and weighted—as key sites of organization. Over time, such stabilized configurations may contribute to the emergence of increasingly integrated levels of organization across scales.

In the next section, we consider the broader implications of this framework, including how signal weighting and coupling contribute to the emergence of structured, adaptive organization across biological systems and beyond.

## Signal weighting enables adaptive organization across scales

Viewing adaptive behavior through the lens of signal weighting provides a way to relate processes that operate across different levels of biological organization. From molecular networks to whole organisms and ecological systems, adaptation depends on the capacity to integrate multiple signals, adjust responses through feedback, and maintain functional stability under changing conditions. By focusing on how relevance is assigned and updated, this framework highlights common organizational features that are not tied to a single scale, mechanism or substrate.

One important implication concerns the emergence of structured organization over time. Adaptive systems are often characterized by increasing levels of integration, in which previously independent components become coordinated through sustained interaction (34). In the context of signal weighting, such integration can be understood as the stabilization of coupled processes that reinforce one another's behavior. Rather than requiring directed progression, this form of organization can arise from the selective retention of interactions that remain coherent under perturbation. In this way, signal weighting offers a perspective on how structured complexity can emerge through local feedback and stabilization, including the integration of previously independent components into coordinated system-level behaviors.

This perspective is also consistent with the view that adaptive organization can extend across biological boundaries. Host–microbiome systems, for example, illustrate how functional units can be distributed across multiple interacting entities. The stability of these systems depends on coordinated signal processing across interfaces, rather than on the properties of any single component (30). Similar principles may apply in ecological systems, where interactions among species contribute to the persistence of community structure despite environmental variability.

At the same time, the logic of signal weighting is not limited to biological substrates. Systems developed in AI provide examples of architectures in which inputs are differentially weighted and updated through feedback (8). In neural networks, for instance, the adjustment of connection strengths determines how signals are propagated and transformed, enabling the system to adapt to patterns in data. While these systems differ fundamentally from biological organisms in their origins and constraints, they share a common operational feature: adaptive behavior emerges through the iterative modification of how signals are weighted within the system.

Considering such parallels does not imply that artificial systems should be classified as living, nor that biological and artificial systems are equivalent. Rather, it suggests that similar organizational principles may underlie adaptive behavior in different contexts. By identifying these shared features, it becomes possible to compare systems that would otherwise be treated as distinct and to ask whether certain properties of adaptation are substrate-independent (see Supplementary Notes S2–S5 for cross-domain instantiations of these principles).

Taken together, these considerations suggest that signal weighting provides a conceptual bridge across domains in which adaptive behavior is observed. By focusing on how systems process and stabilize relevant signals, it becomes possible to relate biological, prebiotic, and artificial systems within a common framework, while preserving the distinctions that arise from their specific implementations.

## Reframing adaptation through signal weighting: toward general principles

The preceding analysis can be distilled into a set of five principles that together describe how adaptive organization emerges through signal weighting. These principles should not be interpreted as an unordered checklist of adaptive properties. Rather, we propose that they define five functionally ordered causal operations through which adaptive systems transform environmental information into coordinated organization. The operations exhibit ordered causal dependencies while participating in a recurrent adaptive architecture, which can be investigated at the level of individual operations, transitions between operations, or the complete adaptive process (Fig. 3).

- Adaptive systems are defined by relevance determination.
- Signal weighting governs the contextual filtering and stabilization of relevant signals.
- Adaptive behavior emerges from the dynamic integration of weighted signals.
- Coupled adaptive systems preferentially retain interactions that reinforce stability, coherence, and persistence.
- Higher-order organization emerges through the recursive stabilization of signal-weighted interactions across interfaces and scales.

**Figure 3:**
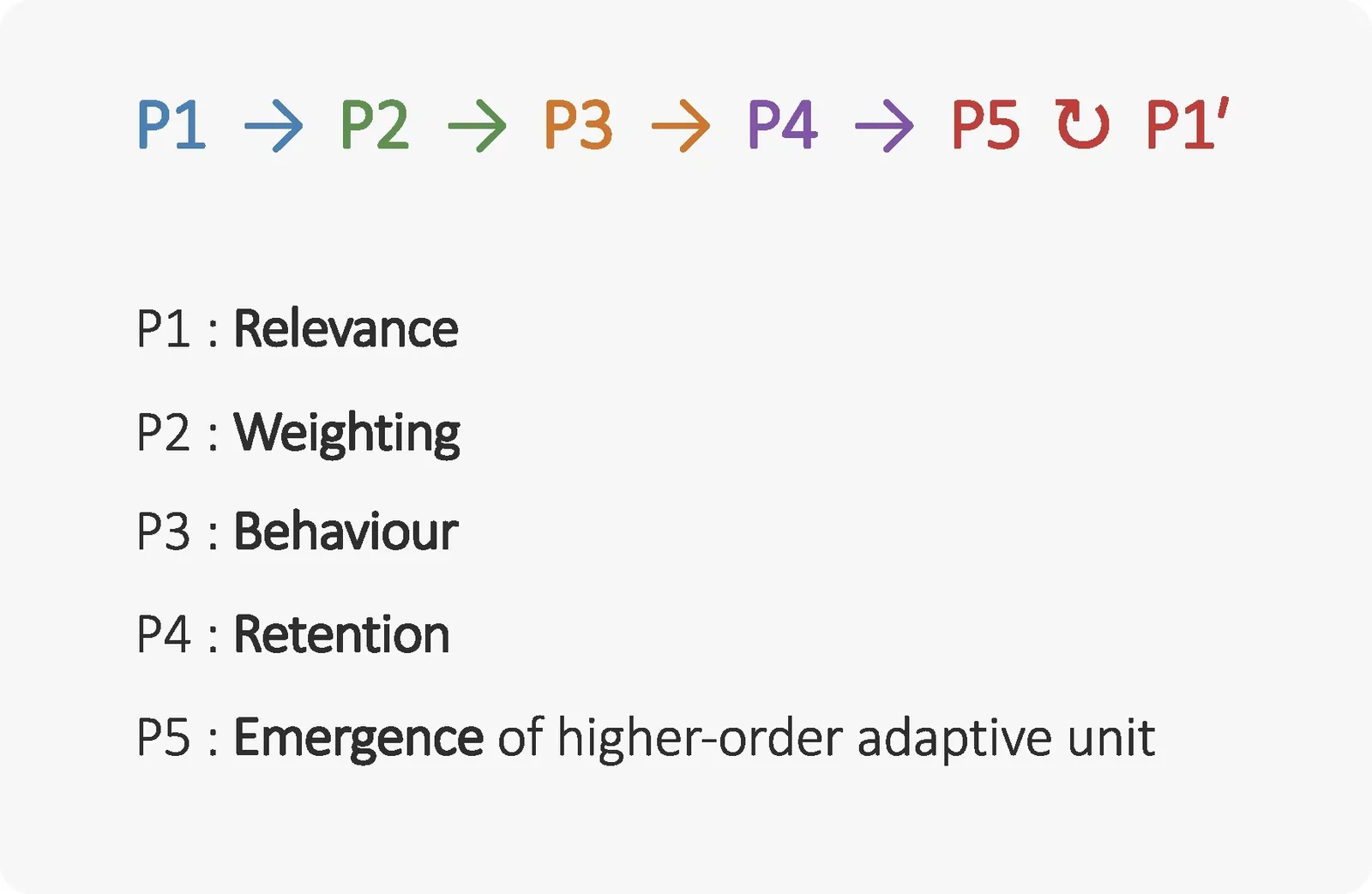
SWT as a recursive causal architecture. The five principles of SWT are proposed as functionally ordered causal operations rather than an unordered set of system properties. Signals first become relevant to an adaptive unit (P1), are differentially weighted according to context (P2), integrated into adaptive behavior (P3), and retained when they stabilize continued function or productive interactions (P4). Repeated stabilization may generate a higher-order adaptive unit (P5). Crucially, formation of a higher-order unit establishes a new system boundary and therefore a new relevance landscape, allowing principle I to be reinstantiated at the higher organizational level (P1′). This recursive architecture allows SWT to be operationalized at multiple levels: individual causal operations, transitions between operations, or the complete adaptive architecture.

The functional ordering of the five principles can be represented as:


Relevance→Weighting→Behaviour→Retention→Higher-orderadaptiveunit

Signals are first delimited as relevant to an adaptive unit (principle I). Relevant signals are then differentially weighted according to context, history, and current state (principle II). Their combined influence gives rise to adaptive behavior (principle III). Behaviors that stabilize continued function or productive interactions are preferentially retained (principle IV). Through repeated stabilization, previously independent components may become sufficiently integrated to function as a higher-order adaptive unit (principle V).

Crucially, principle V does not merely terminate the sequence. The emergence of a higher-order adaptive unit establishes a new system boundary and therefore a new relevance landscape. Signals that were previously relevant to individual components may become buffered, internally regulated or suppressed, while entirely new classes of signals become relevant to the emergent unit. Principle V therefore establishes the conditions under which principle I is reinstantiated at a higher organizational level, allowing the same adaptive architecture to recur across levels of biological organization.

The recursive causal architecture also provides multiple levels of empirical application. Studies may investigate the mechanism implementing a particular operation, test causal transitions between successive operations, or evaluate whether the complete architecture explains adaptive organization better than alternative descriptions. Different biological disciplines may therefore instantiate different parts of the same architecture without requiring identical molecular mechanisms. SWT generates experimentally addressable hypotheses across diverse biological disciplines. Individual operations, their causal transitions, and the complete recursive architecture can therefore be evaluated, refined, or falsified independently as empirical evidence accumulates.

Biology has long sought general principles capable of explaining how living systems organize, persist, and evolve (35). While frameworks centered on replication and selection have been central to this effort, they do not fully capture how adaptive behavior is generated in real time through the interpretation of signals. Across biological systems, from immune recognition to host–microbiome interactions, adaptive responses depend on the continuous assignment and updating of relevance. In this light, signal weighting offers a complementary perspective on the organization of living systems.

By focusing on how signals are filtered, prioritized, and stabilized through feedback, this framework shifts attention from the propagation of variation to the integration of signals at system interfaces. Adaptive systems can be understood as networks that actively construct their behavior by modulating the influence of incoming signals, rather than passively responding to them. When such systems are coupled, their interactions can give rise to stabilized configurations that persist despite ongoing perturbation, supporting the emergence of coordinated organization across scales.

This perspective suggests that directionality in biological systems may arise not only from selection acting on variation but also from the recursive stabilization of interactions that remain coherent over time. In this view, increasing levels of organization reflect the accumulation of compatible signal-weighting processes, rather than the optimization of isolated components. Such an interpretation provides a way to relate phenomena observed across molecular, organismal, and ecological contexts within a common conceptual framework.

More broadly, the logic of signal weighting may extend beyond the boundaries of biology. Systems capable of adapting through feedback-driven modification of signal relevance—whether biological or artificial—share organizational features that can be compared within this framework. While the conditions under which such systems arise differ, the underlying processes by which they stabilize adaptive behavior may follow similar principles.

Taken together, these considerations support the view that adaptive organization may emerge through the dynamic weighting and stabilization of signals across interacting systems. When such processes are coupled, interactions that remain coherent under changing conditions can become preferentially stabilized, contributing over time to increasing levels of coordinated organization, often associated with increasing complexity. By making this logic explicit, signal weighting provides a conceptual bridge across domains and offers a foundation for further investigation into the principles that govern adaptive organization, including the emergence and persistence of living systems. In this light, we propose that signal weighting reflects a general organizational principle observable across well-characterized adaptive systems, and one that may help reconsider how the boundaries of life are defined.

## Supplementary Material

pgag333_Supplementary_Data

## Preprints

This manuscript was posted on a preprint at https://doi.org/10.5281/zenodo.19678448.
