# Supplementary material for: Signal weighting as an organizing principle of adaptive systems
Source: PNAS Nexus. 2026 Oct 1;5(11):pgag333. doi: 10.1093/pnasnexus/pgag333 (PMC13760089; doi:10.1093/pnasnexus/pgag333)
Supplement: Supplementary file 1 [file pgag333_supplementary_data.pdf]

Supplemental Information

Signal weighting as an organizing principle of adaptive systems

Duncan Bruce Sutherland[1] and Bruno Lemaitre[2]

[1] School of Architecture, Civil and Environmental Engineering (ENAC), École Polytechnique Fédérale de Lausanne (EPFL), Lausanne, Switzerland

[2] Global Health Institute, School of Life Sciences, École Polytechnique Fédérale de Lausanne (EPFL), Lausanne, Switzerland

Correspondence: duncan.sutherland@epfl.ch (D.B.S.); bruno.lemaitre@epfl.ch (B.L.)

Table of Contents

Supplemental Information **1**

Signal weighting as an organizing principle of adaptive systems **1**

Supplementary Material Overview **3**

Supplementary Note S1 - Core adaptive architecture of Signal Weighting Theory **4**

S1.1 Minimal recursive causal architecture 4

S1.2 Abstract operators of Signal Weighting Theory 5

S1.3 Recursive hierarchy formation 8

S1.4 Scope, implementation and empirical application 10

S1.5 Relationship to existing theoretical frameworks 13

Supplementary Note S2 - Immunology as real-time adaptive signal weighting **15**

S2.1 Context-dependent signal evaluation in innate immunity 15

S2.2 Innate immune priming 15

S2.3 Adaptive immunity: novelty generation and feedback-driven retention 16

S2.4 Tolerance as weighted interpretation rather than failure of recognition 16

S2.5 Mucosal immunity and IgA as a signal-weighting interface 17

S2.6 Immune memory as retention of interpretive bias 17

S2.7 Summary 17

Supplementary Note S3 - Host-microbiome mutualism and mutualism attractors **20**

S3.1 Bidirectional signalling at the immune-microbiome interface 20

S3.2 IgA as a persistence-biasing interface 20

S3.3 Metabolic feedback and recursive co-adaptation 21

S3.4 Mutualism attractors and early-life tuning 21

S3.5 Cooperative integration and evolutionary consequences 21

S3.6 Summary 22

Supplementary Note S4 - Neural systems as recursive signal-weighting architectures **23**

S4.1 Learning through synaptic weight adjustment 23

S4.2 Attractor dynamics and retention of learned configurations 23

S4.3 Context dependence and graded interpretation 24

S4.4 Coupling across neural systems and collective adaptation 24

S4.5 Scope and limits of the neural analogy 24

S4.6 Summary 25

Supplementary Note S5 - Darwinian evolution as a limiting case of signal weighting **25**

S5.1 Relevance determination in Darwinian evolution 25

S5.2 Externalised signal weighting in Darwinian evolution 26

S5.3 Population-level retention versus real-time interpretation 26

S5.4 Compatibility with SWT and scope of generalisation 27

S5.5 Transition toward internalised weighting 28

S5.6 Summary 28

Supplementary Material Overview

The Supplementary Material provides the formal and biological foundations underpinning Signal Weighting Theory (SWT). Whereas the main Perspective introduces SWT as a minimal recursive causal architecture of adaptation, the Supplement develops the underlying formalism and illustrates how this organisational architecture can be instantiated across diverse adaptive systems.

Supplementary Note S1 introduces the substrate-independent operator formalisation of SWT, defines the five adaptive operations, and relates the recursive architecture to neighbouring theoretical frameworks. **Supplementary Notes S2-S5** then demonstrate how the same organisational architecture is implemented within representative biological domains, including immunology, host-microbiome mutualism, neural systems, and Darwinian evolution.

The purpose of these notes is not to propose a common molecular mechanism or to provide exhaustive reviews of these fields. Rather, they illustrate how distinct biological implementations can instantiate the same adaptive operations while employing different mechanistic substrates, timescales, and levels of biological organisation.

The Supplementary Material should therefore be read as a technical companion to the main manuscript. Together, the operator formalisation, worked biological examples, and illustrative schematics demonstrate how SWT may be interpreted, operationalised, and investigated across diverse adaptive systems.

Supplementary Note S1 - Core adaptive architecture of Signal Weighting Theory

S1.1 Minimal recursive causal architecture

Signal Weighting Theory (SWT) proposes that adaptive systems can be understood through a minimal recursive causal architecture composed of five functionally ordered operations: relevance determination, signal weighting, behavioural integration, retention, and the emergence of higher-order adaptive units. Rather than representing independent system properties or a strictly linear processing sequence, these operations form an ordered set of causal dependencies through which adaptive systems transform available signals into coordinated behaviour and increasingly integrated organisation.

The purpose of this formalisation is not to prescribe specific biological mechanisms, but to identify the minimal causal architecture shared by diverse adaptive systems. Different biological systems - including immune systems, nervous systems, host-microbiome interactions and evolutionary processes - implement these operations through distinct molecular, physiological and ecological mechanisms. SWT therefore operates at the level of organisational architecture rather than mechanistic implementation.

Within this framework, adaptive systems first determine which available signals are relevant to their current state and organisation. Relevant signals are subsequently assigned context-dependent causal influence through signal weighting. Weighted signals are integrated with the current adaptive configuration to generate behaviour. The consequences of behaviour provide feedback that can update adaptive configuration and alter the signals and weighting processes encountered in subsequent adaptive cycles. Behaviours and interactions that reinforce continued function or system coherence are preferentially retained, allowing prior interactions to influence future adaptive responses. Repeated retention of stabilising interactions may ultimately generate higher-order adaptive units that behave as integrated systems in their own right.

A defining feature of SWT is therefore that its causal architecture is recurrent rather than a linear pipeline. Recursion occurs both within adaptive cycles, as behavioural outcomes and retained configurations influence subsequent signal evaluation, and across organisational levels. The emergence of a higher-order adaptive unit establishes a new system boundary and therefore a new relevance landscape. Signals that were previously interpreted by individual components may become internally regulated or buffered, while entirely new classes of signals become relevant to the emergent unit. In this way, higher-order emergence reinstantiates the adaptive architecture in a new organisational context, allowing the same causal architecture to operate repeatedly across increasing levels of biological organisation.

The formalism developed below provides an abstract description of this recursive architecture. It is intentionally substrate-independent and should not be interpreted as a predictive dynamical model. Rather, it specifies the minimal causal operations and dependencies that any implementation of Signal Weighting Theory must instantiate, irrespective of the physical mechanisms through which those operations are realised.

Throughout this Supplementary Information, we use the term *signal* in a broad operational sense. A signal is any time-varying physical, chemical, biological or informational variable capable of influencing the future state or behaviour of an adaptive system. Whether molecular, cellular, physiological, ecological or behavioural, signals need not be intentionally communicated nor possess intrinsic meaning. Rather, their biological significance arises only through the way an adaptive system interprets and differentially responds to them.

S1.2 Abstract operators of Signal Weighting Theory

To formalise the recursive causal architecture proposed by Signal Weighting Theory (SWT), we represent adaptation through five abstract operators corresponding to relevance determination, signal weighting, behavioural integration, retention, and higher-order emergence. These operations are functionally ordered but do not constitute a strictly linear processing chain. Rather, they define a recurrent set of causal dependencies through which adaptive systems evaluate available signals, generate behaviour, update adaptive configuration through feedback, and potentially generate higher-order organisation.

The formalism is intentionally substrate-independent and should therefore be interpreted as an organisational rather than mechanistic description of adaptive systems.

Let S_t denote the set of signals available to an adaptive system at time t. Signals may originate from the external environment, internal physiological states, or interactions with other adaptive systems. Let x_t denote the current system state, h_t accessible interaction history, and C_t the retained adaptive configuration of the system. Whereas h_t captures information from prior interactions that may influence current signal weighting, C_t represents relatively persistent adaptive organisation produced through retention.

Relevance determination

The first operation identifies the subset of available signals that become relevant to the current adaptive unit:

$$S_t^R = \mathcal{R}_{U_n}(S_t, x_t)$$

where $\mathcal{R}_{U_n}$ denotes the relevance operator acting at organisational level n, and S_t^R denotes the subset of available signals that become relevant to adaptive unit U_n. Relevance is therefore not an intrinsic property of a signal itself, but arises from the relationship between available signals, the current state of the system, and its organisation.

Signal weighting

Relevant signals are subsequently assigned context-dependent causal influence:

$$w_t = \mathcal{W}(S_t^R, x_t, h_t)$$

where $\mathcal{W}$ denotes the weighting operator and w_t the resulting vector of signal weights. Weighting therefore depends on the relevant signals, current system state, and accessible interaction history.

Different biological systems implement this operation through diverse mechanisms, including receptor affinities, signalling thresholds, synaptic plasticity, regulatory interactions, ecological feedbacks, or evolutionary selection. Regardless of implementation, weighting determines the relative causal influence of competing signals on subsequent system behaviour.

Behavioural integration

Adaptive behaviour is generated through integration of weighted signals with the retained adaptive configuration of the system:

$$B_t = \mathcal{B}(w_t \odot S_t^R, C_t)$$

where $\mathcal{B}$ denotes the behavioural integration operator, B_t represents the resulting adaptive behaviour, and $\odot$ denotes element-wise weighting of relevant signals.

The inclusion of C_t makes explicit that retained adaptive configuration can influence subsequent behaviour. Thus, identical weighted inputs need not generate identical responses when encountered by systems with different retained configurations. The integration mechanism is intentionally left unspecified, allowing implementation through molecular regulatory networks, neural circuits, physiological regulation, ecological interactions, or other adaptive processes.

Retention

Behaviour generates consequences through interaction with the system's environment or with other systems. We denote feedback arising from these consequences by Φ_t. This feedback is an interaction variable rather than an additional adaptive operator.

Retention updates the adaptive configuration of the system according to its existing configuration, behaviour, and interaction feedback:

$$C_{t+1} = \mathcal{T}(C_t, B_t, \Phi_t)$$

where $\mathcal{T}$ denotes the retention operator. Configurations associated with continued function or coordinated stability are preferentially stabilised, while less effective configurations may decay.

The updated configuration C_{t+1} is therefore not merely an endpoint of the adaptive process. By influencing subsequent behavioural integration, retained configuration provides a causal route through which previous adaptive interactions modify future responses.

Behavioural interaction also modifies the conditions from which subsequent signals arise. This feedback relationship can be represented schematically as:

$$B_t, \Phi_t \rightarrow S_{t+1}$$

Thus, behaviour influences the future signal landscape, allowing the consequences of one adaptive cycle to contribute to the signals available in the next.

Emergence of higher-order adaptive units

Repeated retention of stabilising interactions among coupled adaptive components may generate an integrated adaptive unit operating at the next organisational level:

$$U_{n+1} = \mathcal{E}(C_n^*)$$

where $\mathcal{E}$ denotes the emergence operator, C_n^* represents sufficiently stabilised coupled configurations at organisational level n, and U_{n+1} represents the resulting higher-order adaptive unit.

The emergence of U_{n+1} establishes a new system boundary and associated relevance landscape. The same causal architecture may therefore be instantiated again at the higher organisational level, as developed in Section S1.3.

Causal dependency structure

Although the five SWT operators are functionally ordered, their variables participate in multiple causal dependencies. The core architecture can therefore be summarised schematically as:

$$
\begin{aligned}
&S_t, x_t \rightarrow S_t^R \\
&S_t^R, x_t, h_t \rightarrow w_t \\
&w_t, S_t^R, C_t \rightarrow B_t \\
&B_t, C_t, \Phi_t \rightarrow C_{t+1} \\
&B_t, \Phi_t \rightarrow S_{t+1} \\
&C_n^* \rightarrow U_{n+1}
\end{aligned}
$$

This dependency structure makes explicit that SWT is not a linear pipeline. Signals participate in both relevance determination and subsequent weighting and integration; retained adaptive configuration influences future behaviour; and behaviour modifies the conditions from which subsequent signals arise. The resulting architecture is therefore recurrent and history-dependent while retaining a functional ordering among the five adaptive operations.

Importantly, these operators should not be interpreted as biochemical mechanisms, optimisation algorithms, or predictive dynamical equations. Rather, they define minimal causal operations and dependencies that organise adaptive behaviour independently of the physical processes through which they are realised. Different biological systems may therefore instantiate the same organisational architecture through entirely different molecular, physiological, ecological, or computational mechanisms. SWT consequently compares adaptive systems at the level of causal organisation rather than mechanistic implementation.

S1.3 Recursive hierarchy formation

A defining feature of Signal Weighting Theory is that the emergence of a higher-order adaptive unit does not terminate adaptive organisation but establishes the conditions under which the same causal architecture may operate at a new organisational level. Once sufficiently stabilised, an emergent adaptive unit acquires its own organisational boundary, functional identity, and capacity to interact with its environment. Consequently, the signals

that become relevant to the newly formed unit may differ from those that were relevant to its constituent components.

Following the notation introduced in Section S1.2, this recursive transition can be expressed conceptually as

$$U_{n+1} = \mathcal{E}(C_n^*)$$

followed by

$$U_{n+1} \rightarrow \mathcal{R}_{U_{n+1}}$$

where C_n^* represents sufficiently stabilised coupled configurations at organisational level n, $\mathcal{E}$ denotes the emergence operator, and U_{n+1} denotes the resulting higher-order adaptive unit. The formation of U_{n+1} establishes a new organisational context in which relevance is determined relative to the properties and boundary of the emergent unit.

Higher-order emergence therefore changes the space of signals capable of influencing adaptive behaviour. Signals previously interpreted directly by lower-level components may become internally coordinated, buffered, or suppressed, while new environmental or system-level signals become relevant to the emergent unit. Principle V is consequently not simply an endpoint of the preceding adaptive operations. It provides the transition through which stabilised lower-level organisation can become the substrate for a new instantiation of the adaptive architecture.

This recursion operates across organisational levels and is distinct from the within-cycle recurrence described in Sections S1.1 and S1.2. Within an adaptive unit, behaviour and its consequences modify subsequent signal conditions and retained adaptive configuration. Across levels, sufficiently stabilised coupled configurations may generate a new adaptive unit, for which relevance determination, signal weighting, behavioural integration, and retention operate in a new organisational context. The causal architecture can therefore recur without requiring identical mechanisms or behaviours at successive levels.

Each newly formed adaptive unit may consequently possess a distinct relevance landscape, weighting mechanisms, behavioural repertoire, and retention processes. The organisational architecture remains conserved at the level of causal relations, while its physical implementation can change across scales. Molecular networks, cells, multicellular organisms, host-microbiome systems, social groups, and other higher-order adaptive entities may therefore be analysed as candidate instantiations of the same underlying adaptive

architecture, without implying that the mechanisms producing or maintaining these systems are equivalent.

This recursive organisation has a useful conceptual connection to renormalisation-group (RG) approaches. RG theory provides a general mathematical framework for relating descriptions across scales, commonly through coarse-graining in which lower-scale degrees of freedom are integrated into effective higher-scale descriptions [1,2]. Principle V of SWT is structurally compatible with this perspective insofar as stabilised interactions among lower-level components can be represented as an effective higher-order unit with a new set of relevant variables. However, SWT does not assume that higher-order adaptive organisation constitutes an RG transformation, nor does the emergence operator $\mathcal{E}$ specify a coarse-graining procedure. Rather, SWT identifies the causal-organisational condition of interest: the point at which a stabilised higher-order configuration itself participates in relevance determination, signal weighting, behaviour and retention. RG and related coarse-graining methods may therefore provide mathematical approaches for investigating particular scale transitions proposed within SWT [1,2]. In SWT, emergence therefore denotes not merely a change in descriptive scale, but the establishment of a higher-order adaptive unit with a new relevance landscape - a new locus of adaptive relevance.

The recursive relationship described above therefore does not simply repeat individual operations. It reinstantiates the complete adaptive architecture in a new organisational context. In this sense, Principle V establishes the conditions under which Principle I can operate at the next level:

$$\boxed{U_n \xrightarrow{\mathcal{R},\mathcal{W},\mathcal{B},\mathcal{T}} C_n^* \xrightarrow{\mathcal{E}} U_{n+1} \xrightarrow{\mathcal{R}_{U_{n+1}}} \cdots}$$

Adaptive complexity may therefore increase through repeated instantiation of the same minimal causal architecture across successive organisational levels, without requiring the introduction of new causal principles at each level.

S1.4 Scope, implementation and empirical application

The formalism presented above specifies the minimal causal architecture proposed by Signal Weighting Theory rather than a predictive dynamical model or a specific mechanistic implementation. Consequently, SWT should be interpreted as a framework for analysing adaptive organisation across diverse biological systems rather than as a replacement for established mechanistic theories within individual disciplines.

The recursive organisational architecture formalised in the preceding sections is summarised schematically in Supplementary Figure S1. The figure illustrates the five functionally ordered

adaptive operations proposed by Signal Weighting Theory and their recursive organisation across scales. Within an adaptive unit, behavioural consequences modify subsequent signal conditions and retained adaptive configuration, while the emergence of a higher-order adaptive unit establishes a new relevance landscape in which the same architecture may be instantiated again.

The abstract operators defined in Section S1.2 may be instantiated through a wide variety of physical mechanisms. These operations are typically instantiated in biological systems through distributed architectures that transform incoming signals into context-dependent outputs, rather than through single components corresponding one-to-one with individual operators (see Supplementary Notes S2-S5). Representative examples are summarised in Supplementary Table S1, illustrating how identical adaptive operations may be realised through fundamentally different biological processes. In immune systems, weighting may be implemented through receptor affinities, signalling thresholds, and feedback between immune cells and microbial communities. In neural systems, weighting may arise through synaptic plasticity and network dynamics. In ecological systems, weighting may emerge through changing interaction strengths among organisms, while in Darwinian evolution weighting is largely externalised through differential persistence across generations. Although these mechanisms differ fundamentally, they can be analysed using the same underlying causal architecture.

This organisational perspective allows SWT to be operationalised at multiple levels of investigation.

Node level. Individual mechanisms may be examined according to the adaptive operation they implement, such as relevance determination, signal weighting, behavioural integration, retention, or emergence.

Transition level. Experimental perturbations may investigate how changes in one adaptive operation influence causally dependent variables or operations within the recurrent architecture. For example, altering signal weighting should predictably modify behavioural integration; modifying retained adaptive configuration should alter subsequent behavioural responses; and disrupting retention should influence the stability of higher-order adaptive organisation. Likewise, experimentally altering behavioural outputs may test whether resulting changes in system-environment interaction modify the subsequent signal landscape.

Architecture level. Entire adaptive systems may be analysed to determine whether the five operations and their recurrent causal dependencies better explain observed organisation than alternative theoretical descriptions. This level addresses how coordinated adaptive behaviour emerges from the interaction of the complete architecture rather than from any individual component in isolation.

By distinguishing organisational architecture from mechanistic implementation, SWT provides a common conceptual language for comparing adaptive systems across biological scales while remaining compatible with established mechanistic theories within individual disciplines. The formalism therefore serves as a general framework for identifying where adaptive processes operate within a causal architecture, irrespective of the molecular, physiological, ecological, or evolutionary mechanisms through which those processes are realised.

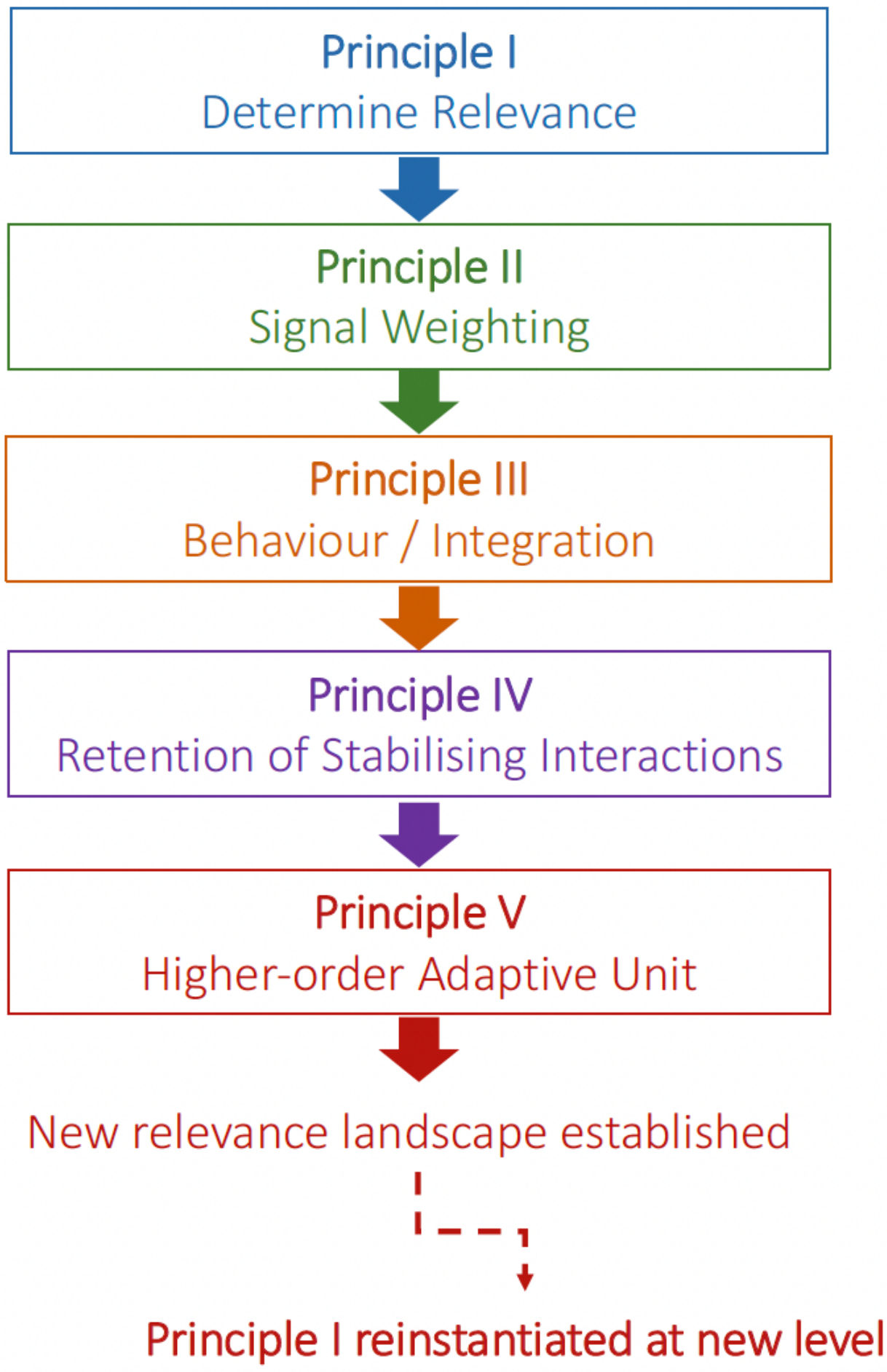

Supplementary Figure S1. Recursive operational architecture of Signal Weighting Theory (SWT). The figure illustrates the five functionally ordered adaptive operations formalised in Supplementary Note S1. Adaptive systems determine the relevance of available signals (Principle I), assign context-dependent causal influence through signal weighting (Principle II), integrate weighted signals with retained adaptive configuration to generate behaviour (Principle III), and preferentially retain configurations associated with continued function or coordinated stability (Principle IV). Behavioural consequences modify subsequent signal conditions and retained adaptive configuration, producing recurrence within an adaptive unit. Repeated stabilisation of coupled interactions may generate higher-order adaptive units (Principle V), establishing new system boundaries and relevance landscapes in which the same architecture may be instantiated again. The accompanying operator formalisation defines these adaptive operations independently of any particular biological, computational, or mathematical implementation.

Adaptive operation	Abstract operator	Biological implementation
Relevance	Identify relevant inputs	receptor specificity, microbial identity, sensory filtering
Weighting	Assign context-dependent influence	affinity, co-stimulation, neuromodulation
Behaviour	Generate adaptive output	immune response, movement, gene regulation
Retention	Stabilise beneficial interactions	Immune memory, interface stabilization, synaptic consolidation, ecological selective retention
Higher-order emergence	Form new adaptive unit	multicellular tissues, host-microbiome partnerships, eusocial colonies

Supplementary Table S1. Mapping between adaptive operations, abstract operators, and representative biological implementations. Representative examples illustrating how the abstract adaptive operations defined by Signal Weighting Theory may be instantiated through diverse biological mechanisms. While the underlying molecular, physiological, ecological and evolutionary processes differ across domains, each implements the same organisational operation within the recursive adaptive architecture proposed by Signal Weighting Theory. The examples are illustrative rather than exhaustive.

S1.5 Relationship to existing theoretical frameworks

Numerous theoretical frameworks address aspects of adaptive behaviour, including dynamical systems theory, control theory, efficient coding, predictive processing, reinforcement learning and the Free Energy Principle, together with domain-specific biological frameworks spanning evolution, development, immunology and ecology[3–10]. These approaches have generated important mechanistic insights into adaptive behaviour. However, they generally address particular mechanisms or organisational levels rather than asking whether a common minimal causal architecture underlies how adaptive systems determine relevance, integrate signals, stabilise interactions and generate higher-order organisation across biological domains.

Several existing theoretical frameworks already treat adaptive behaviour as the outcome of context-dependent integration of information, particularly within neuroscience and theoretical biology. Predictive coding, Kalman filtering, precision-weighted predictive

processing, and the Free Energy Principle all describe mechanisms by which systems infer unobserved (hidden) states from sensory inputs, update beliefs or minimise prediction error through weighted information processing [7–10]. Signal Weighting Theory is intended to complement rather than replace these approaches. Rather than beginning with inference or prediction, SWT asks a more general organisational question: how does an adaptive system determine which signals should differentially influence subsequent behaviour?

Kalman filtering provides a well-established engineering example in which the relative influence of incoming observations is continuously adjusted through the Kalman gain[7]. Although originally developed for optimal state estimation rather than biological adaptation, it illustrates how context-dependent weighting of information can be formalised within a specific mathematical framework.

These examples illustrate how diverse theoretical frameworks implement context-dependent information weighting through specific computational or mathematical mechanisms. Signal Weighting Theory instead seeks to identify the minimal organisational operations common to these diverse implementations.

Supplementary Note S2 - Immunology as real-time adaptive signal weighting

This note illustrates how the five adaptive operations defined in Supplementary Note S1 are instantiated within the immune system.

S2.1 Context-dependent signal evaluation in innate immunity

The immune system provides a well-characterised example of a biological system capable of real-time adaptive interpretation of signals. Innate immune receptors such as Toll-like receptors (TLRs) detect conserved molecular motifs associated with microbes or tissue damage. However, receptor engagement alone rarely determines immune outcome. Identical ligands can induce tolerance, inflammation, or repair depending on the context in which signals are received [11].

Signal interpretation in innate immunity depends on multiple factors, including co-receptor engagement, signalling thresholds, metabolic state, tissue environment, and the history of prior stimulation. These factors modulate downstream pathways, allowing the same molecular cue to be weighted differently under different conditions. As a result, innate immune responses are graded and conditional rather than binary, reflecting an underlying architecture in which signals are evaluated relative to internal state and contextual constraints [12].

From an SWT perspective, innate immunity implements contextual signal weighting: inputs are filtered through internal parameters that determine their functional significance, enabling flexible and adaptive responses to a noisy and variable environment.

S2.2 Innate immune priming

Innate immune priming provides a clear illustration of recursive signal weighting in immunological systems. Following an initial exposure, subsequent encounters can elicit faster and stronger responses, a phenomenon described as innate immune memory or reactivation-based memory [11]. From an SWT perspective, this behaviour reflects the retention of prior signal-weighting configurations, which bias future interpretation and response. Rather than encoding fixed responses, the immune system retains an updated interpretive state that modulates how subsequent signals are evaluated.

This form of history-dependent modulation illustrates how adaptive directionality can emerge within the lifetime of a system through feedback-driven updating, without requiring population-level selection.

S2.3 Adaptive immunity: novelty generation and feedback-driven retention

Adaptive immunity introduces an additional layer of recursive signal weighting through the generation and selective retention of novel antigen receptors. B cells diversify their antigen-binding receptors via somatic hypermutation, a process initiated by activation-induced cytidine deaminase (AID) [13]. This mechanism generates a continual stream of receptor variants that far exceeds what could be specified directly by the germline genome.

Crucially, this novelty is not retained indiscriminately. Within germinal centres, B-cell clones undergo structured competition in which survival and expansion depend on the strength and quality of signals received through antigen binding, T-cell help, and co-stimulatory pathways [14–16]. Feedback from these interactions selectively stabilises receptor configurations that perform well under prevailing conditions.

This process exemplifies recursive adaptive updating. Variation is generated locally and continuously; evaluation occurs through structured feedback; and successful configurations are retained in memory compartments. The result is not a fixed instruction set, but a history-dependent interpretive bias that shapes future immune responses. Adaptive immunity therefore operates as a learning system, in which internal weights are updated in response to interaction outcomes rather than predetermined rules.

S2.4 Tolerance as weighted interpretation rather than failure of recognition

An essential feature of immune adaptation is the capacity to interpret signals in a manner that favours tolerance rather than activation. T-cell receptor signalling operates across graded thresholds, with co-inhibitory pathways, cytokine milieu, and metabolic cues determining whether encounters lead to effector differentiation, anergy, or regulatory phenotypes [17].

These tolerance mechanisms demonstrate that immune meaning is not encoded in ligand identity alone. Instead, meaning emerges from integration across multiple weighted inputs, including tissue context, microbial metabolites, and prior exposure history. Metabolites derived from commensal microbes, such as short-chain fatty acids, further modulate signalling thresholds, linking immune interpretation to local ecological and energetic conditions [18].

From an SWT standpoint, tolerance reflects a regime in which certain signal-response configurations are preferentially retained because they stabilise system coherence over time. Rather than representing a failure of detection, tolerance is an actively learned outcome of recursive signal weighting.

S2.5 Mucosal immunity and IgA as a signal-weighting interface

At mucosal surfaces, the signal-weighting logic of immunity is particularly evident. Secretory IgA, the dominant antibody isotype in the intestine, undergoes continuous diversification and updating, enabling fine-grained regulation of microbial communities [14]. Rather than functioning solely as a neutralising effector, IgA modulates microbial behaviour, spatial organisation, and ecological niches [15,19].

IgA coating influences microbial access to nutrients, proximity to the epithelium, and interaction with other microbes, thereby shaping community structure without sterilisation [20,21]. In SWT terms, IgA acts as a contextual weighting interface, biasing microbial persistence and behaviour in ways that stabilise cooperative configurations.

Microbial metabolites, in turn, feed back into immune regulation, reinforcing IgA production and tolerance pathways[22]. This bidirectional loop couples immune interpretation to microbial activity, enabling host and microbiota to co-adjust over time. Stability in this system arises not from exclusion, but from mutual adaptive weighting across an interface.

The gut-associated lymphoid architecture provides a representative implementation of the SWT architecture, transforming microbial and host-derived inputs through context-dependent immune selection and integration into a dynamically regulated IgA repertoire that, in turn, modifies subsequent host-microbial signalling.

S2.6 Immune memory as retention of interpretive bias

Immune memory encodes the outcomes of past signal-weighting processes rather than fixed responses to specific stimuli. Memory B and T cells retain biases shaped by prior encounters, enabling faster and more effective interpretation upon re-exposure [23]. Importantly, these biases are modifiable: memory responses can be reshaped by new contexts, antigenic variation, or altered ecological conditions.

This flexibility distinguishes immune memory from static instruction sets. Retention is conditional and adaptive, allowing the immune system to remain responsive while preserving coherence across time. Such memory illustrates how history-dependent weighting enables adaptive directionality without requiring foresight or central control.

S2.7 Summary

Taken together, immunological systems exemplify a fully realised instantiation of recursive signal weighting. Signals are evaluated contextually, internal parameters are updated through feedback, and successful interpretive configurations are selectively retained. The immune

system does not merely recognise patterns; it learns how to interpret them in ways that stabilise organismal integrity and ecological coexistence.

As such, immunology provides a mechanistically grounded example of how adaptive meaning, tolerance, and cooperative stability can emerge through recursive interpretation - core features formalised in Signal Weighting Theory.

Collectively, these observations illustrate how the five adaptive operations formalised in Supplementary Note S1 are implemented within the immune system. Relevance determination, context-dependent signal weighting, behavioural integration, retention, and the emergence of increasingly coordinated adaptive organisation are realised through immunological mechanisms rather than through the abstract operators themselves. Supplementary Figure S2 provides a representative implementation of this architecture at the IgA-microbiome interface, illustrating how each abstract adaptive operator corresponds to specific immunological mechanisms and their resulting adaptive outputs.

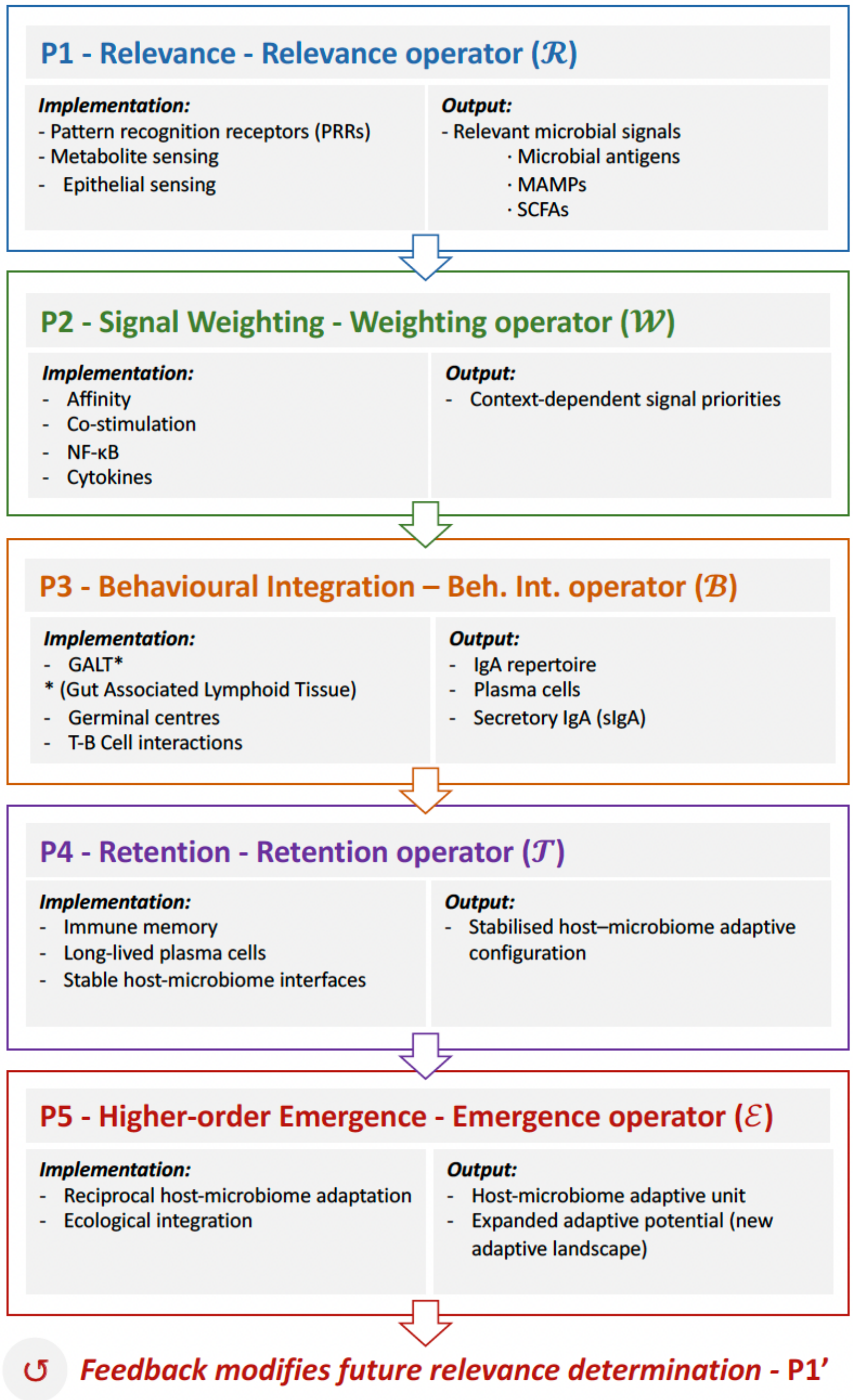

Supplementary Figure S2. Operational implementation of Signal Weighting Theory at the IgA-microbiome interface. Representative implementation of the recursive adaptive architecture shown in Supplementary Figure S1. Microbial signals are first selected through receptor-mediated relevance determination (Principle I), assigned context-dependent influence (Principle II), integrated through mucosal immune networks to generate IgA responses (Principle III), stabilised through immune memory and persistent host-microbiome interactions (Principle IV), and ultimately contribute to a higher-order host-microbiome adaptive unit (Principle V). The emergence of this higher-order adaptive unit establishes a new relevance landscape within which the adaptive architecture can be reinstantiated, allowing subsequent host-microbiome interactions to be evaluated in the context of the emergent unit.

Supplementary Note S3 - Host-microbiome mutualism and mutualism attractors

This note illustrates how the adaptive architecture defined in Supplementary Note S1 is distributed across host-microbiome interactions.

S3.1 Bidirectional signalling at the immune-microbiome interface

The intestinal microbiome represents a paradigmatic case in which adaptive stability emerges through continuous bidirectional signalling between interacting systems. Host tissues, immune cells, and microbial communities exchange chemical, metabolic, and immunological signals that are evaluated and weighted on both sides of the interface. These signals do not simply trigger fixed responses but are interpreted relative to ecological context, developmental history, and system state.

Microbial metabolites such as short-chain fatty acids, bile acid derivatives, and tryptophan metabolites influence epithelial function, immune differentiation, and metabolic regulation [24–26]. Conversely, host-derived signals - including antimicrobial peptides, IgA, mucus composition, and epithelial patterning - shape microbial spatial organisation and growth dynamics. Stability in this system depends on how these signals are integrated and recursively adjusted, rather than on unilateral control by either partner.

From an SWT perspective, the host-microbiome interface functions as a distributed signal-weighting architecture in which adaptive bias emerges through reciprocal feedback.

S3.2 IgA as a persistence-biasing interface

Seminal work in mucosal immunology established that IgA is not merely a neutralising antibody but a regulator of microbial behaviour and ecological positioning [19,15,21]. IgA coating influences microbial access to nutrients, adherence to epithelial surfaces, susceptibility to exclusion, and participation in microbial consortia. These effects allow IgA to bias microbial persistence without eliminating diversity.

In SWT terms, IgA acts as a persistence-biasing interface. Rather than selecting microbes solely based on replication speed or antigenicity, IgA specificity, abundance, and coating patterns bias microbial persistence by preferentially stabilising taxa and behaviours compatible with host physiology. Microbes that integrate effectively into host metabolism and immune tolerance are preferentially stabilised, while destabilising expansion is constrained.

This mode of regulation differs from classical selection acting solely through differential replication and elimination. Retention is assigned at the level of interaction patterns, not individual replicators, and operates continuously rather than through episodic elimination.

S3.3 Metabolic feedback and recursive co-adaptation

Microbial metabolites feed back into immune and epithelial systems, reinforcing tolerance pathways and IgA production. Short-chain fatty acids, for example, promote regulatory immune phenotypes (including Treg differentiation) and epithelial integrity, which in turn stabilise microbial niches that support their production [22]. This establishes a recursive feedback loop in which host and microbial activities co-adjust over time.

Such coupling enables fine-grained adaptation to environmental changes such as diet, infection, or developmental transitions. Importantly, adaptive responses emerge from mutual signal weighting, not from optimisation by either system in isolation. The host does not simply impose constraints on microbes, nor do microbes passively exploit host resources. Instead, stability arises through ongoing negotiation mediated by weighted signals.

S3.4 Mutualism attractors and early-life tuning

These dynamics can give rise to mutualism attractors: relatively stable configurations in which host and microbial activities reinforce one another's persistence. Mutualism attractors are not fixed end states but dynamically maintained regimes shaped by early-life exposure, immune learning, diet, and environmental perturbations.

Early developmental windows are particularly important for attractor formation. Initial microbial colonisation, immune education, and metabolic context bias how signals are weighted later in life, influencing susceptibility to inflammatory disease, metabolic instability, or resilience [27,28]. Perturbations such as antibiotics or nutritional disruption can destabilise established attractors, leading to shifts toward less cooperative configurations [29,30].

From an SWT perspective, mutualism attractors represent basins of retention in the space of possible host-microbe interactions, stabilised through recursive signal weighting rather than through genetic fixation.

S3.5 Cooperative integration and evolutionary consequences

Host-microbiome mutualism illustrates how adaptive systems can increase their functional capacity by acting on each other's behalf. By sustaining microbial partners that expand metabolic flexibility, immune calibration, and environmental responsiveness, the host effectively extends its adaptive repertoire beyond what could be achieved by its own genome alone.

This cooperative integration has evolutionary consequences. Systems capable of stabilising mutualistic interactions can explore broader ecological niches, buffer environmental variability, and support energy-intensive life-history traits. In SWT terms, such systems increase their interpretive depth, enabling more nuanced and resilient engagement with their environment.

These observations challenge views of adaptation that focus exclusively on individual-level optimisation. Instead, they highlight how evolutionary directionality can emerge through the stabilisation of cooperative configurations across interacting systems.

S3.6 Summary

Host-microbiome systems exemplify a mode of adaptation in which stability and directionality arise from recursive signal weighting across interfaces rather than arising solely from differential replication efficiency among independently replicating taxa. IgA-mediated regulation, metabolic feedback, and immune tolerance together implement a distributed adaptive architecture that selectively retains cooperative interaction patterns.

Mutualism attractors capture the outcome of this process: dynamically maintained configurations in which persistence is assigned to interactions that enhance system-level coherence. These dynamics provide a concrete biological instantiation of the principles formalised in Signal Weighting Theory, illustrating how cooperative stability and adaptive expansion can emerge through recursive interpretation and retention.

Host-microbiome mutualism demonstrates that the same five adaptive operations can be distributed across interacting biological systems. Here, adaptive organisation emerges through reciprocal signalling and stabilisation across the host-microbiome interface, illustrating how the common organisational architecture of SWT can be implemented through ecological interactions.

Supplementary Note S4 - Neural systems as recursive signal-weighting architectures

This note illustrates how the organisational operations of SWT are implemented in neural systems.

S4.1 Learning through synaptic weight adjustment

Neural systems provide a well-studied example of adaptive behaviour in which changes in effective connection strengths provide a useful computational abstraction for recursive signal weighting. Classical theories of learning, beginning with Hebb's proposal that correlated neuronal activity modifies subsequent interactions, inspired later mathematical and computational formulations in which adaptive behaviour emerges through iterative adjustment of connection weights [31–34]. From the perspective of Signal Weighting Theory (SWT), these connection weights provide an illustrative abstraction of internal parameters that modulate how incoming signals influence future network dynamics.

Importantly, SWT does not propose that biological learning is reducible to synaptic weight modification alone. Neural adaptation is supported by multiple interacting mechanisms, including synaptic and intrinsic neuronal plasticity, structural remodelling, neuromodulatory systems, glial interactions, and other forms of circuit reorganisation.[33]. Rather, the language of weight updating is used here as a computational abstraction that captures the organisational logic of recursive adaptation across diverse biological implementations. Accordingly, the discussion below should be interpreted at the level of organisational architecture rather than as a comprehensive account of the molecular mechanisms underlying neuronal plasticity.

S4.2 Attractor dynamics and retention of learned configurations

Recursive weight updating in neural systems can give rise to stable patterns of activity known as attractor states [32]. Attractors correspond to internally stabilised configurations toward which network dynamics converge, enabling reliable recall, pattern completion, and decision-like behaviour.

Attractor formation illustrates how retention operates in neural systems. Rather than storing information as explicit symbols, networks retain successful interpretive configurations by stabilising weight patterns that reliably generate coherent responses. These configurations persist across time and bias future signal interpretation, functioning as memory [32].

In SWT terms, attractors represent retained signal-weight configurations that have proven effective in prior interactions. Importantly, they are not immutable: new experiences can

reshape or destabilise attractors through further recursive updating, preserving flexibility while maintaining coherence.

S4.3 Context dependence and graded interpretation

Neural responses are intrinsically context dependent. The same sensory input can produce different outputs depending on network state, neuromodulatory signals, and recent history. Graded firing thresholds, inhibitory circuits, and modulatory neurotransmitters adjust how signals are weighted across neural populations [35].

This context sensitivity parallels immune signal interpretation and host-microbiome regulation. Meaning is not intrinsic to the input signal but emerges from distributed integration across weighted pathways. Neural systems therefore exemplify how adaptive meaning arises relationally, as an emergent property of system state shaped by prior feedback.

S4.4 Coupling across neural systems and collective adaptation

Neural systems do not operate in isolation. In social organisms, brains continuously exchange structured signals through communication, shared environments, and coordinated behaviour. Developmental contexts such as the mother-infant dyad provide especially dense feedback loops in which neural states co-adapt through repeated interaction [36,37].

Such coupling allows recursive signal weighting to extend across systems, enabling alignment, synchronisation, and the emergence of shared behavioural patterns. At a collective level, this can give rise to social learning, coordination, and distributed problem solving - properties that cannot be reduced to individual neural dynamics alone.

From an SWT perspective, these phenomena reflect interaction-level retention, in which patterns of coordination are selectively stabilised through mutual feedback across coupled systems.

S4.5 Scope and limits of the neural analogy

Neural systems are not privileged examples within SWT, nor does SWT claim that all adaptive systems function like brains. The relevance of neural networks lies in the explicit measurability of weighting and update dynamics, which make recursive adaptation experimentally accessible.

Neural learning illustrates one realisation of recursive signal weighting among many. Its value for SWT is therefore operational rather than foundational: it demonstrates how adaptive

directionality, memory, and context-dependent interpretation can arise from weight updating without reliance on genetic inheritance or population-level selection.

S4.6 Summary

Neural systems instantiate recursive signal weighting through synaptic plasticity, attractor formation, and context-dependent integration of signals. Adaptive behaviour emerges as weight configurations are updated and selectively retained based on experience, biasing future interpretation.

These dynamics parallel those observed in immune systems and host-microbiome interactions, despite profound differences in mechanism and scale. Neural systems thus provide an independent biological domain in which the adaptive logic formalised by Signal Weighting Theory can be directly observed and measured.

Neural systems provide an independent implementation of the five adaptive operations described in Supplementary Note S1. Although realised through neuronal plasticity and network dynamics rather than immune or ecological mechanisms, the underlying organisational architecture remains the same.

Supplementary Note S5 - Darwinian evolution as a limiting case of signal weighting

This note illustrates how the adaptive operations defined in Supplementary Note S1 are implemented through population-level evolutionary processes.

S5.1 Relevance determination in Darwinian evolution

In physiological adaptive systems, relevance is determined through receptors and signalling interfaces that selectively identify information requiring adaptive response. In Darwinian evolution, relevance is not established through real-time physiological evaluation within an individual organism. Instead, ecological conditions determine which organism-environment interactions become consequential for persistence and reproduction. Environmental change therefore defines the dimensions along which phenotypic variation becomes relevant. Differential survival and reproduction subsequently assign greater adaptive influence to these relevant interactions through population-level differential retention across generations. Thus, the organisational operations described by SWT remain preserved, while their biological implementation differs fundamentally from real-time physiological adaptation.

A classic example is provided by Darwin's finches. During periods of drought, the predominance of large, hard seeds renders beak morphology a relevant ecological variable.

Individual finches do not determine this relevance physiologically; rather, the altered environment establishes which organism-environment interactions become consequential for persistence and reproduction. Differential survival and reproduction subsequently increase the representation of phenotypes with more suitable beak morphologies over successive generations. Within SWT, relevance is therefore established externally by ecological conditions, while weighting is implemented retrospectively through differential retention across generations.

S5.2 Externalised signal weighting in Darwinian evolution

Darwinian evolution provides one of the most familiar and successful frameworks for explaining adaptive change in biological systems. At its core, the Darwinian process operates through three elements: variation, heredity, and differential persistence [38,39]. From the perspective of Signal Weighting Theory (SWT), this process can be understood as a limiting case of recursive signal weighting, in which weighting and retention are externalised to the environment and distributed across populations rather than implemented within individual systems.

In classical natural selection, heritable variation introduces differences among individuals, while environmental conditions act as a filtering process that determines which variants persist. In SWT terms, phenotypic variation corresponds to a space of possible responses to environmental signals, while differential survival and reproduction correspond to the selective retention of certain signal-response configurations over others. The “weights” that determine which variants persist are not adjusted internally by the organism in real time, but are instead imposed retrospectively by population-level outcomes [40].

This externalisation distinguishes Darwinian evolution from adaptive systems that interpret and weight signals during their own lifetimes. Natural selection assigns persistence after the fact, operating on realised phenotypes rather than on ongoing interpretive processes within the organism.

S5.3 Population-level retention versus real-time interpretation

In Darwinian evolution, the environment functions as a global evaluator that differentially retains variants based on their consequences for survival and reproduction. The feedback loop that biases future system behaviour operates across generations: variants that persist shape the composition of subsequent populations, thereby influencing the distribution of traits exposed to future selective pressures.

This temporal structure contrasts with systems that perform real-time signal weighting, such as immune or neural systems, in which interpretation and feedback occur within the lifetime of the system itself. In SWT terms, Darwinian evolution involves slow, coarse-grained

recursive updating, with long delays between variation, evaluation, and retention. The system does not adjust its own internal parameters in response to signals; instead, unsuccessful configurations are eliminated, and successful ones are reproduced.

Seen this way, Darwinian evolution implements a form of signal weighting in which:

- signals correspond to environmental conditions,
- responses correspond to phenotypic traits,
- weights are effectively binary (persist or disappear),
- updates occur only through differential reproduction.

This mode of weighting is powerful but limited in speed and flexibility.

S5.4 Compatibility with SWT and scope of generalisation

Interpreting Darwinian evolution as a limiting case of signal weighting does not diminish its explanatory power. Rather, it clarifies its scope conditions. Natural selection excels at explaining long-term adaptive change across populations, but it is less suited to describing systems that must respond flexibly to fluctuating conditions on short timescales.

SWT generalises adaptive logic by allowing weighting, feedback, and retention to occur:

- within individual systems (for example, immune or neural learning within a lifetime),
- across interacting systems (such as host-microbiome mutualism or social communication),
- and on timescales shorter than generational turnover (for instance, physiological regulation or behavioural adaptation).

Darwinian evolution occupies one end of this continuum, providing an instructive limiting case for understanding how the organisational architecture of adaptation can be implemented through different biological mechanisms.

Under this view, Darwinian evolution represents a regime in which recursive signal weighting is implemented predominantly through population dynamics rather than through real-time adaptive updating within individual organisms. More complex adaptive systems embed signal weighting internally, enabling learning, tolerance, anticipation, and cooperative integration without waiting for generational replacement.

Within Signal Weighting Theory, Darwinian evolution represents a limiting case in which the organisational operation of signal weighting is preserved while its implementation differs fundamentally from that observed in physiological adaptive systems. Rather than assigning context-dependent influence through real-time modulation of signals within an individual

organism, Darwinian evolution externalises the weighting process through differential survival and reproduction across generations. Environmental conditions therefore influence future adaptive organisation retrospectively through the differential persistence of variants that remain functionally coherent under prevailing conditions.

This distinction illustrates the broader principle underlying SWT: identical organisational operations need not be implemented through identical biological mechanisms. Immune systems implement weighting through receptor signalling and feedback, neural systems through activity-dependent plasticity, host-microbiome systems through reciprocal ecological feedback, whereas Darwinian evolution implements weighting through population-level differential retention across generations. SWT therefore compares adaptive systems at the level of organisational architecture rather than mechanistic implementation.

S5.5 Transition toward internalised weighting

Biological evolution itself has progressively incorporated internal signal-weighting mechanisms. Regulatory networks, developmental plasticity, immune learning, and neural adaptation all introduce layers of within-lifetime interpretation that complement and accelerate population-level selection. These mechanisms do not replace Darwinian evolution but operate alongside it, reshaping how organisms engage with selective pressures.

From an SWT perspective, this trajectory reflects a shift from external to internal weighting, in which adaptive bias is increasingly implemented through real-time interpretation rather than post hoc elimination. Such internalisation allows systems to stabilise favourable configurations more rapidly, explore cooperative interactions, and retain adaptive structure without relying solely on reproductive filtering.

S5.6 Summary

Darwinian evolution can thus be understood as a special case of adaptive signal weighting in which relevance is established by ecological conditions, weighting is externalised through differential survival and reproduction, and retention occurs through population-level persistence across generations. SWT does not challenge this framework, but extends it by identifying a broader class of adaptive architectures in which weighting and retention operate not only within individual systems, but also across interfaces between interacting systems, and when such couplings stabilise, through cooperative integration into higher-order functional units.

This reinterpretation preserves the central insights of evolutionary theory while clarifying how adaptive directionality can emerge in systems that learn, tolerate, and coordinate in real time - features that are difficult to capture using population-level selection alone.

Darwinian evolution illustrates that the five adaptive operations may also be implemented across populations and generations. Here, relevance is established by ecological conditions, while weighting and retention are externalised through differential survival and reproduction, further illustrating that SWT compares adaptive systems at the level of organisational architecture rather than mechanistic implementation.

Acknowledgments

We thank Florent Masson and Thomas Pradeu for insightful discussions on conceptual frameworks of biological organization. D.B.S. also thanks Christian Ludwig for discussions on chemical biasing processes at interfaces. We thank Orson Sutherland for helpful discussions and comments on the systems-level formalisation of the framework. D.B.S. further acknowledges Sidonia Fagarasan and Hiroshi Ohno for intellectual guidance in microbiome science that informed aspects of this work. B.L. acknowledges support from the Swiss National Science Foundation (SNSF; grant 310030_215073). This manuscript was posted on a preprint: https://doi.org/10.5281/zenodo.19678448.

Declaration of Interests

D.B.S. is a founder of Arkaiya SA. The authors declare no other competing interests.

Data Availability

There are no data underlying this work.

References:

1. Kadanoff, L. P. Scaling laws for ising models near T c. *Physics Physique Fizika* **2**, 263–272 (1966).
2. Wilson, K. G. Renormalization Group and Critical Phenomena. I. Renormalization Group and the Kadanoff Scaling Picture. *Phys. Rev. B* **4**, 3174–3183 (1971).
3. Waddington, C. H. Canalization of Development and the Inheritance of Acquired Characters. *Nature* **150**, 563–565 (1942).
4. Noble, D. A theory of biological relativity: no privileged level of causation. *Interface Focus.* **2**, 55–64 (2012).
5. Lewontin, R. C. The Units of Selection. *Annu. Rev. Ecol. Syst.* **1**, 1–18 (1970).
6. Kitano, H. Biological robustness. *Nat Rev Genet* **5**, 826–837 (2004).
7. Kalman, R. E. A New Approach to Linear Filtering and Prediction Problems. *Journal of Basic Engineering* **82**, 35–45 (1960).
8. Rao, R. P. N. & Ballard, D. H. Predictive coding in the visual cortex: a functional interpretation of some extra-classical receptive-field effects. *Nat Neurosci* **2**, 79–87 (1999).
9. Feldman, H. & Friston, K. J. Attention, Uncertainty, and Free-Energy. *Front. Hum. Neurosci.* **4**, (2010).
10. Friston, K. A free energy principle for a particular physics. Preprint at https://doi.org/10.48550/ARXIV.1906.10184 (2019).
11. Pradeu, T., Thomma, B. P. H. J., Girardin, S. E. & Lemaitre, B. The conceptual foundations of innate immunity: Taking stock 30 years later. *Immunity* **57**, 613–631 (2024).
12. Medzhitov, R. Origin and physiological roles of inflammation. *Nature* **454**, 428–435 (2008).

13. Honjo, T., Kinoshita, K. & Muramatsu, M. Molecular mechanism of class switch recombination: linkage with somatic hypermutation. *Annual Review of Immunology* **20**, 165–196 (2002).

14. Fagarasan, S. & Honjo, T. Intestinal IgA synthesis: regulation of front-line body defences. *Nature Reviews Immunology* **3**, 63–72 (2003).

15. Kawamoto, S. *et al.* The inhibitory receptor PD-1 regulates IgA selection and bacterial composition in the gut. *Science* **336**, 485–489 (2012).

16. Victora, G. D. & Nussenzweig, M. C. Germinal centers. *Annu Rev Immunol* **30**, 429–457 (2012).

17. Sakaguchi, S. *et al.* Immunologic tolerance maintained by CD25+ CD4+ regulatory T cells: their common role in controlling autoimmunity, tumor immunity, and transplantation tolerance. *Immunol Rev* **182**, 18–32 (2001).

18. Furusawa, Y. *et al.* Commensal microbe-derived butyrate induces the differentiation of colonic regulatory T cells. *Nature* **504**, 446–450 (2013).

19. Fagarasan, S. *et al.* Critical roles of activation-induced cytidine deaminase in the homeostasis of gut flora. *Science* **298**, 1424–1427 (2002).

20. Fagarasan, S., Kawamoto, S., Kanagawa, O. & Suzuki, K. Adaptive Immune Regulation in the Gut: T Cell–Dependent and T Cell–Independent IgA Synthesis. *Annu. Rev. Immunol.* **28**, 243–273 (2010).

21. Nakajima, A. *et al.* IgA regulates the composition and metabolic function of gut microbiota by promoting symbiosis between bacteria. *J Exp Med* **215**, 2019–2034 (2018).

22. Takeuchi, T. *et al.* Acetate differentially regulates IgA reactivity to commensal bacteria. *Nature* **595**, 560–564 (2021).

23. Ahmed, R. & Gray, D. Immunological Memory and Protective Immunity: Understanding Their Relation. *Science* **272**, 54–60 (1996).

24. Koh, A., De Vadder, F., Kovatcheva-Datchary, P. & Bäckhed, F. From Dietary Fiber to Host Physiology: Short-Chain Fatty Acids as Key Bacterial Metabolites. *Cell* **165**, 1332–1345 (2016).

25. Hang, S. *et al.* Bile acid metabolites control TH17 and Treg cell differentiation. *Nature* **576**, 143–148 (2019).

26. Zelante, T. *et al.* Tryptophan Catabolites from Microbiota Engage Aryl Hydrocarbon Receptor and Balance Mucosal Reactivity via Interleukin-22. *Immunity* **39**, 372–385 (2013).

27. Gensollen, T., Iyer, S. S., Kasper, D. L. & Blumberg, R. S. How colonization by microbiota in early life shapes the immune system. *Science* **352**, 539–544 (2016).

28. Tamburini, S., Shen, N., Wu, H. C. & Clemente, J. C. The microbiome in early life: implications for health outcomes. *Nat Med* **22**, 713–722 (2016).

29. Cox, L. M. *et al.* Altering the Intestinal Microbiota during a Critical Developmental Window Has Lasting Metabolic Consequences. *Cell* **158**, 705–721 (2014).

30. David, L. A. *et al.* Diet rapidly and reproducibly alters the human gut microbiome. *Nature* **505**, 559–563 (2014).

31. Hebb, D. O. *The Organization of Behavior: A Neuropsychological Theory*. (Wiley, New York, 1974).

32. Hopfield, J. J. Neural networks and physical systems with emergent collective computational abilities. *Proc. Natl. Acad. Sci. U.S.A.* **79**, 2554–2558 (1982).

33. Holtmaat, A. & Svoboda, K. Experience-dependent structural synaptic plasticity in the mammalian brain. *Nat Rev Neurosci* **10**, 647–658 (2009).

34. Richards, B. A. *et al.* A deep learning framework for neuroscience. *Nat Neurosci* **22**, 1761–1770 (2019).

35. Friston, K. The free-energy principle: a unified brain theory? *Nat Rev Neurosci* **11**, 127–138 (2010).

36. Hasson, U., Ghazanfar, A. A., Galantucci, B., Garrod, S. & Keysers, C. Brain-to-brain coupling: a mechanism for creating and sharing a social world. *Trends in Cognitive Sciences* **16**, 114–121 (2012).

37. Feldman, R. Parent-infant synchrony and the construction of shared timing; physiological precursors, developmental outcomes, and risk conditions. *J Child Psychol Psychiatry* **48**, 329–354 (2007).

38. Darwin, C. *On the Origin of Species*. (John Murray, 1859).

39. Maynard Smith, J. *Evolution and the Theory of Games*. (Cambridge University Press, 1982).

40. Fisher, R. A. *The Genetical Theory of Natural Selection*. (Clarendon Press, 1930).
